# Compatible interface design of CoO-based Li-O_2_ battery cathodes with long-cycling stability

**DOI:** 10.1038/srep08335

**Published:** 2015-02-27

**Authors:** Chaoqun Shang, Shanmu Dong, Pu Hu, Jing Guan, Dongdong Xiao, Xiao Chen, Lixue Zhang, Lin Gu, Guanglei Cui, Liquan Chen

**Affiliations:** 1 Qingdao Industrial Energy Storage Research Institute, Qingdao Institute of Bioenergy and Bioprocess Technology, Chinese Academy of Sciences, Qingdao 266101, P. R. China; 2 University of Chinese Academy of Sciences, Beijing 100049, P. R. China; 3 Institute of Physics, Chinese Academy of Sciences, Beijing 100080, P. R. China

## Abstract

Lithium-oxygen batteries with high theoretical energy densities have great potential. Recent studies have focused on different cathode architecture design to address poor cycling performance, while the impact of interface stability on cathode side has been barely reported. In this study, we introduce CoO mesoporous spheres into cathode, where the growth of crystalline discharge products (Li_2_O_2_) is directly observed on the CoO surface from aberration-corrected STEM. This CoO based cathode demonstrates more than 300 discharge/charge cycles with excessive lithium anode. Under deep discharge/charge, CoO cathode exhibited superior cycle performance than that of Co_3_O_4_ with similar nanostructure. This improved cycle performance can be ascribed to a more favorable adsorption configuration of Li_2_O_2_ intermediates (LiO_2_) on CoO surface, which is demonstrated through DFT calculation. The favorable adsorption of LiO_2_ plays an important role in the enhanced cycle performance, which reduced the contact of LiO_2_ to carbon materials and further alleviated the side reactions during charge process. This compatible interface design may provide an effective approach in protecting carbon-based cathodes in metal-oxygen batteries.

Lithium-Oxygen (Li-O_2_) batteries with high theoretical gravimetric energy densities are the most tantalizing energy storage devices^1^,^2^,^3^,^4^. However, low round-trip efficiency and poor cycle life caused by high overpotential during charge process limit their practical application^5^,^6^,^7^,^8^. One of the most important reasons responsible for the irreversible side reaction is the instability of carbon-based cathode^9^,^10^,^11^,^12^. Up to now, carbon materials have been intensively studied as the basis of cathode for Li-O_2_ batteries due to its high electronic conductivity, low cost and porous structure^13^,^14^,^15^. Unfortunately, it suffers from the formation of Li_2_CO_3_ at the carbon/electrolyte or carbon/Li_2_O_2_ interface^7^,^16^. Carbon decomposition can be mainly attributed to the reaction with Li_2_O_2_ intermediates (generally proposed as LiO_2_), forming Li_2_CO_3_ during charge process^10^,^11^,^17^. Therefore, it is necessary to reduce this side reaction by introducing a favorable component into carbon based cathodes^13^,^18^,^19^.

Among various options, Co-based transition metal oxides may be promising candidates. Co_3_O_4_ was reported as a promoter to enhance the interfacial transport of LiO_2_, which imply that the favorable interaction between LiO_2_ and Co-based oxides may protect carbon materials from oxidative decomposition during charge process^20^. Although substantial recent studies on cobalt-based oxide materials have exhibited better cycle performance, most of them attribute the improved cycle performance to the conventional electrocatalytic activity and the nanostructure of cathodes^9^,^21^,^22^,^23^,^24^,^25^. Up to date, there is still rare report concerning the adsorption configuration of Co-based oxides with LiO_2_ as a factor for enhancing the cycle performance^26^.

In this paper, CoO mesoporous spheres were investigated as cathode catalysts in Li-O_2_ batteries. The CoO-based carbon cathodes (denoted as CoO cathodes) performed significantly enhanced stability, which cycled 30 times under unrestrained capacity with high capacity retention. The contribution of adsorption configuration of CoO with LiO_2_ was investigated by density functional theory (DFT) calculations. Corroborated by electrochemical performance, we propose that the enhanced cycle performance of CoO materials can be attributed to the favorable adsorption configuration of LiO_2_ on CoO. While the importance of nanostructure fabrication for cathode has been extensively highlighted in recent studies, this study provides an insight into adsorption configuration impact on the overall electrochemical performance Li-O_2_ batteries.

## Results

### Characterization of as-prepared CoO mesoporous spheres

**Figure 1:**
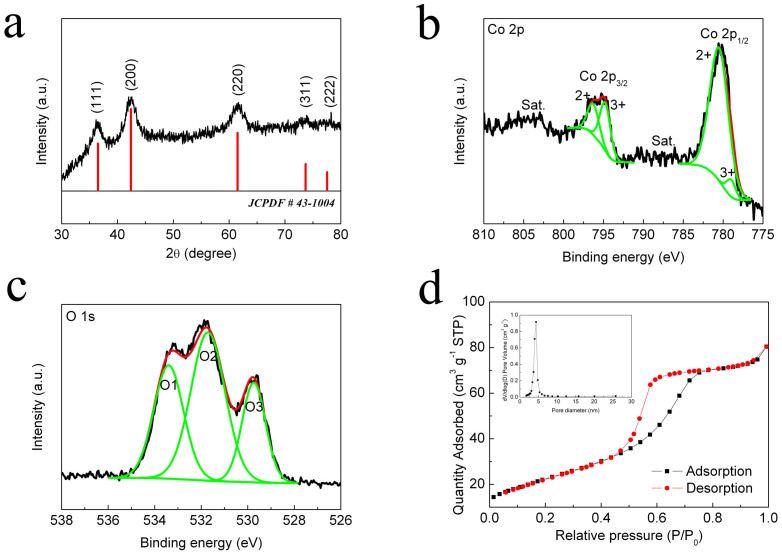
(a) XRD patterns of as-prepared CoO. (b) XPS spectrum of Co 2p. (c) XPS spectrum of O 1s. (d) Nitrogen adsorption and desorption isotherms of as-prepared CoO and pore-size distribution (inset).

The X-ray diffraction (XRD) patterns (Fig. 1a) revealed that all the diffraction peaks could be indexed to CoO (JCPDF: 43-1004) and the broad peaks indicated the low crystallinity^27^,^28^. The grain size of CoO was estimated to be approximately 14.8 nm as calculated from the Scherrer equation applied to the (111), (200) and (220) peaks. And the lattice parameter calculated from the as-mentioned three peaks was about 4.26 Å. XPS was further used to characterize more detailed elemental composition of the as-prepared CoO. The Co 2p spectrum was fitted with Co^2+^ as shown in Fig. 1b. For O 1s spectrum (Fig. 1c), there were three contributions denoted as O1, O2 and O3, respectively. The component O1 at 533.4 eV was attributed to the multiplicity of physi-sorbed and chemi-sorbed water on or near the surface which confirmed the large surface area of as-prepared CoO. The O2 at 531.7 eV was usually associated with oxygen in OH^−^ groups. And the O3 sitting at 529.7 eV was typical of metal-oxygen bonds^29^. The CoO spheres hold a large specific surface area of 82.15 m^2^ g^−1^, which is confirmed by the nitrogen absorption-desorption isotherms (Fig. 1d). This would offer a large electrode-electrolyte interface to ensure favorable accessibility of the active sites as well as storage space for insoluble discharge products^30^. The average pore size of CoO was approximately 4.5 nm (inset of Fig. 1d), which facilitated the accessible diffusion of electrolytes.

**Figure 2:**
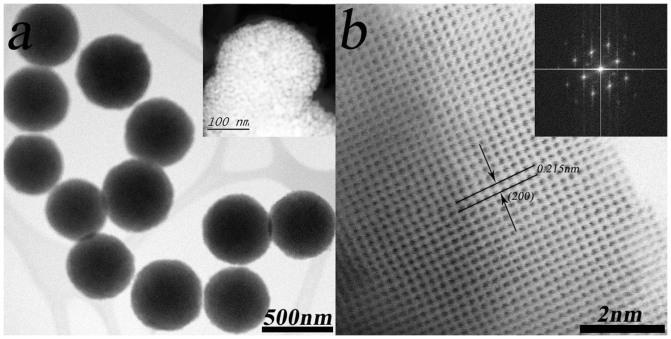
Typical TEM image (a) and STEM image (b) of CoO mesoporous spheress.

Aberration-corrected STEM was carried out to characterize the microscopic morphology of CoO. The STEM images of Fig. 2a manifested CoO with well defined mesoporous spheres ranging from 250 to 450 nm. It was worth noting that these CoO mesoporous spheres were consisted of rough aggregated single-crystalline nanoparticles (inset of Fig. 2a). The spacing of the lattice fringes was calculated to be 0.215 nm, which could be indexed as the (200) planes of cubic CoO (JCPDF: 43-1004). The selected area electron diffraction (SAED) patterns for the CoO mesoporous spheres (inset of Fig. 2b) clearly demonstrated the well-textured and single-crystalline nature of primary nanoparticles. This well defined nanostructure and single crystal nature of primary particles made it easier for direct visual identification of discharge products accumulation on CoO surfaces (further discussed below).

### Electrochemical performance of CoO cathodes

**Figure 3:**
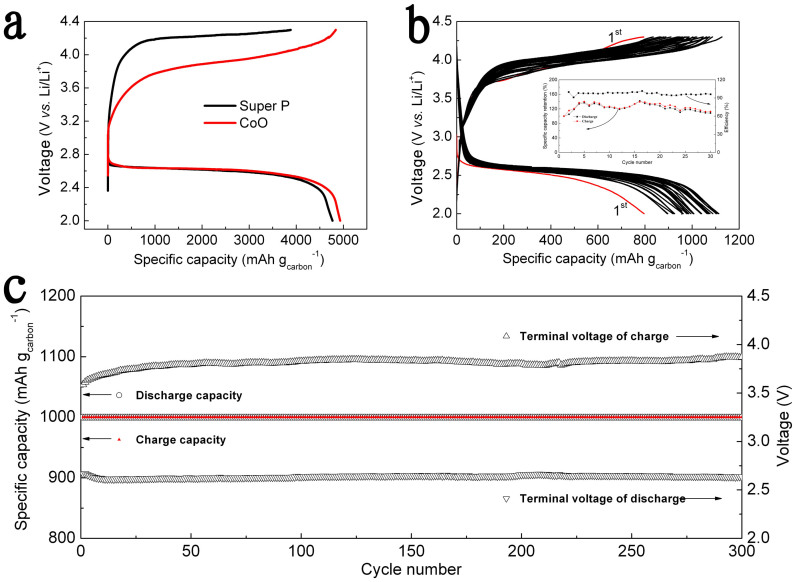
(a) The first galvanostatic cycle of Super P (black) and CoO (red) at a current density of 0.04 mA cm^−2^ with 1 M LiTFSI/TEGDME electrolytes under saturated O_2_. (b) The deep discharge-charge cycling performance and coulombic efficiency (inset) of CoO cathodes Li-O_2_ battery. (c) The cycling performance of CoO cathodes Li-O_2_ battery at the current density of 0.04 mA cm^−2^ with fixed discharge-charge capacities of 1000 mAh g_carbon_^−1^.

The first discharge-charge curves of the Li-O_2_ batteries at a current density of 0.04 mA cm^−2^ were illustrated in Fig. 3a to investigate the role of CoO on the oxygen reduction reaction (ORR) and oxygen evolution reaction (OER) kinetics. The CoO cathodes (typically 1 mg cm^−2^) delivered a discharge specific capacity of 4849 mAh g_carbon_^−1^ and a charge specific capacity of 4840.5 mAh g_carbon_^−1^, indicating high coulombic efficiency of 99.8% for the first cycle. It should be noted that all the capacities are based on the mass of carbon materials (Super P) in the electrodes. The discharge voltage of CoO (~ 2.6 V) was similar to that of Super P, while the charge plateau of CoO (3.75 V) was lower than that of Super P (~ 4.25 V) by 500 mV. It should be noting that the charge potential of CoO was still high, which was similar with previous report of Co_3_O_4_^16^. Here we did not state that CoO is necessarily an electrocatalyst to lower the activation energy through electron transfer. In contrast to traditional electron-transfer catalysts in Li-O_2_ batteries to lower charge overpotential, we proposed that CoO may alleviate the contact of LiO_2_ with carbon materials, which would reduce the formation of Li_2_CO_3_ and further result in long cycle stability (further discussed below). As shown in Fig. 3b, CoO cathodes (typically 4 mg cm^−2^) performed excellent stability with unrestrained capacity at a current density of 0.04 mA cm^−2^. For the initial 5 cycles, the discharge capacity increased step by step. This phenomenon was caused by the gradual penetration of electrolyte into the mesoporous CoO cathodes with large surface area^20^. For the following cycles, the capacity was relatively stable. After 30 cycles, the capacity retention of CoO cathodes obviously excelled the performance of Super P cathodes (only 10% capacity retention for 10 cycles) as illustrated in Fig. S1. Fig. 3c displayed the cycling stability of the Li-O_2_ battery with CoO cathodes at a cutoff capacity of 1000 mAh g_carbon_^−1^. The specific capacity presented no decay over 300 cycles, which implied that CoO cathodes were highly stable with sustained performance (further discussed below). All these results indicated that CoO mesoporous spheres were potential cathode materials for non-aqueous Li-O_2_ battery.

## Discussion

**Figure 4:**
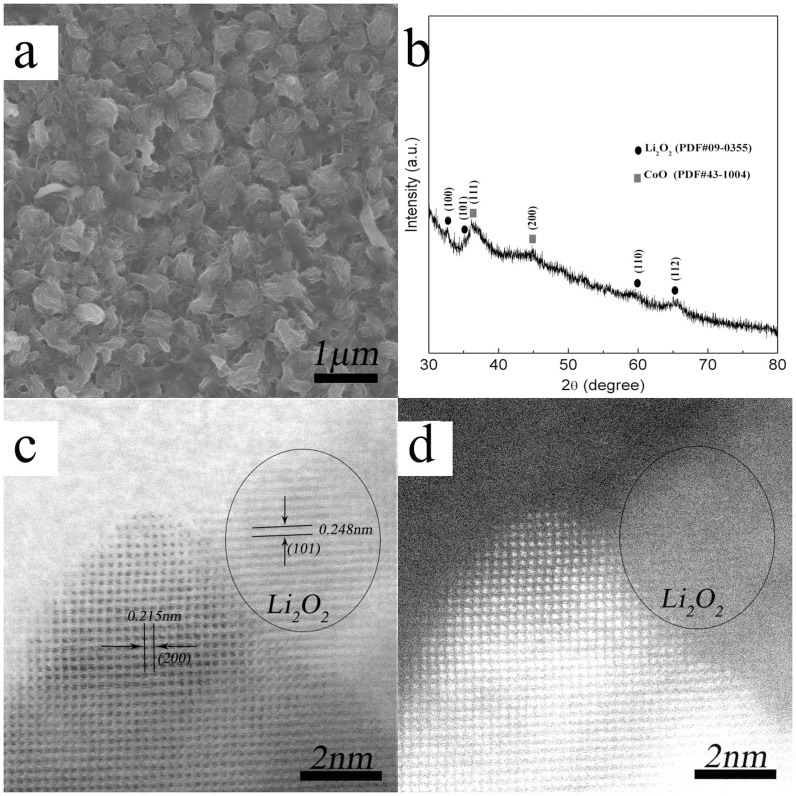
(a) The typical SEM image of CoO cathodes after full discharge at current density of 0.04 mA cm^−2^. (b) XRD patterns of CoO cathodes at the end of the first full discharge until 2.0 V at current density of 0.04 mA cm^−2^ (Because of the poor cystallinity of Li_2_O_2_, The CoO-based paste was directly coated on glass fiber as the cathodes to avoid the strong influence of nickel foam.). (c) Typical annular bright-field and (d) corresponding high-angle annular dark-field images of Li_2_O_2_ on CoO.

The morphology of discharge products was investigated by SEM imaging. As shown in Fig. 4a, the CoO cathodes after the first full discharge were covered by toroid-like particles with rough surface. The discharge products were identified as Li_2_O_2_ through XRD analysis (Fig. 4b). The peaks labeled with circles were reasonably assigned as the (100), (101) and (110) peaks of Li_2_O_2_ (JCPDF 09-0355), while others matched well to the XRD pattern of CoO (JCPDF: 43-1004). The Li_2_O_2_ peaks were so weak in the XRD spectrum, indicating a lower degree of Li_2_O_2_ crystallinity^5^,^30^,^31^,^32^. To further confirm the presence of Li_2_O_2_, aberration-corrected STEM was performed after the first discharge. There was a region that was only found on the annular-bright-field image (Fig. 4c) but not on the corresponding high-angle annular-dark-field image (Fig. 4d), suggesting the presence of light element only. The lattice fringes of 0.248 nm was corresponding to the (101) space of Li_2_O_2_^32^,^33^,^34^. Interestingly, Li_2_O_2_ particles directly observed by aberration-corrected STEM were formed on CoO surface. This result may indicate the presence of electronic conductivity pathways in Li_2_O_2_ formed on CoO surface. This surface electronic conductivity could play a role in the reversible formation and decomposition of Li_2_O_2_. Therefore, it may also imply that CoO cathodes could obtain a promising cycle performance with more recharged discharge products^35^,^36^. Furthermore, the well defined morphology and single-crystalline nature of CoO particles were easy to discriminate from Li_2_O_2_ with poor crystallinity. It was worth noting that the lattice fringes of (200) plane of CoO did not change at all after full discharge, demonstrating that CoO was a stable component in the cathodes.

**Figure 5:**
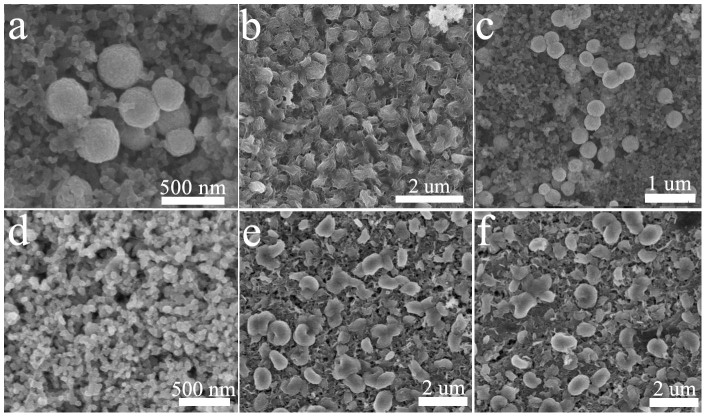
The typical SEM images of CoO cathodes and Super P cathodes at the pristine (a, d), first discharged (b, e) and first charged (c, f) states.

CoO cathodes exhibited more stable performance than Super P cathodes (as compared above). To evaluate the superior coulombic efficiency of CoO cathodes, *ex situ*-SEM was used to observe the change of cathodes after the first discharge and charge as displayed in Fig. 5. Before discharge, the CoO mesoporous spheres and Super P nanoparticles were homogeneously distributed (Fig. 5a). After the first discharge, toroidal-like Li_2_O_2_ particles with rough surface were observed at CoO cathodes (Fig. 5b), which was different from the morphology of Li_2_O_2_ formed at Super P cathodes (Fig. 5e). The unsmooth surface of toroidal particles implied a low crystallinity of Li_2_O_2_ formed on CoO cathodes. After charge, the toroidal particles decomposed completely (Fig. 5c), indicating the reversible formation/decomposition of Li_2_O_2_. However, Li_2_O_2_ particles with smooth surface still remained on the Super P cathodes (Fig. 5f), which caused the poor coulombic efficiency (81.4%, Fig. 3a). The gases generated in the charging processes of the Li-O_2_ cells with CoO and Super P cathodes were subjected to Gas Chromatography-Mass Spectrum (GC-MS), respectively. As presented in Table 1, the presence of CoO lowers the proportion of CO_2_ that was produced by Li_2_CO_3_ and electrolyte decomposition above 4 V. This further indicated the importance of CoO in charge process. The discharge profile of CoO cathodes was similar to that of Super P cathodes (Fig. 3a), revealing a comparable ORR ability of both cathodes to form toroidal Li_2_O_2_ (Fig. 5b and 5e). But during charge process, CoO impacted the OER distinctly as the CoO cathodes delivered a lower charge plateau and higher coulumbic efficiency than that of Super P cathodes.

The CoO cathodes Li-O_2_ battery didn't work any more after 80 cycles (Fig. S2), which was considered ‘failed’. The battery was subsequently dissected and was found still saturated with electrolyte, thus demonstrating that the exhaustion of electrolyte was not the main reason of the battery failure in our case. On the other hand, shiny lithium anode disappeared completely; whereas white powder generated. The lithium in the anode was found to have limited reversibility that the thickness of LiOH layer increased steadily as the cycling progressed according to a recent report^37^, where the deactivation of carbon-black cathode (clogged by discharge products) caused the failure of battery although the anode was still functioning. Unlikely, the failure of CoO-based battery was mainly attributed to the complete consumption of lithium foil (diameter 10 mm; thickness 0.2 mm). The purpose of this paper was to demonstrate the potential of using CoO as cathodes in Li-O_2_ battery rather to present a finally practical and commercial solution. Herein, another CoO battery was built by introducing thicker lithium foil (diameter 10 mm; thickness 2 mm) to provide enough lithium. In the following tests, the new battery with excess lithium demonstrated excellent cycling performance for more than 300 cycles, which further indicated the dramatic role of CoO in impacting the discharge products. Besides, the benefit of Li-O_2_ batteries was based on the assumption that lithium was completely reversible during the discharge-charge process. It was noted that the use of a protected lithium anode in a practical battery to mitigate the sacrifice of lithium was indispensable.

**Figure 6:**
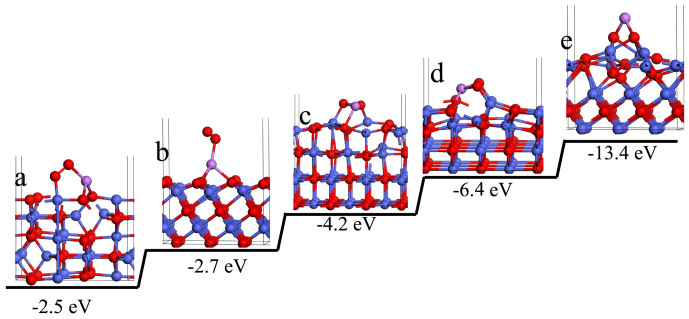
Side views of the most stable adsorption configurations of LiO_2_ on (a) Co_3_O_4_ (110); (b) O-CoO (111); (c) CoO (200); (d) CoO (220) and (e) Co-CoO (111) surfaces.

Additionally, Co_3_O_4_ mesoporous spheres were also investigated as the cathodes that delivered moderate performance (Fig. S3). In consideration of the similar nanostructure of CoO and Co_3_O_4_, the superior performance of CoO cathodes might be attributed to intrinsic surface configuration. Since LiO_2_ was demonstrated to have some solubility in electrolyte^5^,^20^,^38^, moderating the distribution of LiO_2_ during charge process may be critical to the stability of carbon materials. And alleviating the contact of LiO_2_ with carbon may be a direct way to reduce the formation of Li_2_CO_3_^16^,^20^. Therefore, we proposed that the adsorption configuration of LiO_2_ on CoO would have a more favorable impact on alleviating this side reaction and further improving the cycle performance. Density functional calculations (DFT) were employed to investigate the adsorption configuration of LiO_2_ on CoO and Co_3_O_4_, respectively. Fig. 6c showed that LiO_2_ species formed three connections with the CoO (200) surface, which was corresponding to the oxygen end of LiO_2_ located on top of Co^2+^ sites and Li formed bond with two equivalent O^2−^ sites so that the (O-Li-O-O-Co) adopted a distorted pentagonal structure. The resultant adsorption energy of this state was −4.2 eV, which was much stronger than that of carbon materials (−0.1 eV) as well as that of Co_3_O_4_ (110) surface (−2.5 eV, more details in Supporting Information). During charge process, LiO_2_ was prior adsorbed to CoO, which would prevent the formation of Li_2_CO_3_ effectively. In further DFT calculations, we found that CoO exhibited much higher adsorption energy for each facet (Fig. 6b–e). The strong adsorption of CoO to LiO_2_ played a key role in the cycling stability, which would more effectively reduce the possibility of LiO_2_ contacting to carbon and consequently alleviate the formation of Li_2_CO_3_.

## Conclusions

In summary, CoO mesoporous spheres with well defined nanostructure performed excellent stability for Li-O_2_ battery tests, which cycled 30 times under unrestrained capacity without considerable capacity fades. To clarify the impact of adsorption configuration, we demonstrated that CoO cathodes delivered superior cycle performance than that of Co_3_O_4_ with the same nanostructure. DFT was introduced to investigate the adsorption properties of LiO_2_ on the experimentally realized CoO, Co_3_O_4_ and carbon materials. The more stable adsorption configuration of LiO_2_ on CoO played a dominant role in the enhanced cycle performance, which reduced the contact of LiO_2_ to carbon materials and further alleviated the side reactions during charge process. The design of a cathode with well matched adsorption configuration of LiO_2_ was expected to guide further Li-O_2_ battery development.

## Methods

### Preparation of CoO

All the chemicals were used directly without further purification. In a typical synthesis, 2 mmol cobalt nitrate hexahydrate was dissolved in 30 mL n-hexanol to form a red solution. 0.8 g of F-127 was added to the red solution with continuous stirring and a translucent solution was obtained after 20 min. Then the solution was transferred to a 50 mL Teflon-lined autoclave, followed by solvothermal treatment at 180°C for 5 h. After cooled down to room temperature, the dark gray precursor was harvested by filtration and washed with deionized water and ethanol before drying at 80°C overnight. Co_3_O_4_ was obtained by heating the precursor under air at 280°C for 300 min at a heating rate of 5°C min^−1^. Co_3_O_4_ was further annealed at 200°C in a flowing Ar/H_2_ (95:5) for 2 h with a progressive, slow heating ramp (1°C min^−1^). After cooled down, CoO mesoporous sphere was finally obtained. The nanowire CoO was collected from below steps. 1.16 g Co(NO_3_)_2_·6H_2_O and 1.2 g CO(NH_2_)_2_ were dissolved in 50 mL water under stirring for 20 min and further transferred into autoclave. Then the autoclave was sealed and maintained at 110°C for 8 h. After cooled down, the resultant precursor was managed with the same calcination steps to CoO mesoporous sphere.

### Characterizations

X-ray diffraction (XRD) pattern was recorded in a Bruker-AXS Micro-diffractometer (D8 ADVANCE) from 10 to 85°. X-ray photoelectron spectroscopy (XPS) was carried out by an ESCALab220i-XL electron spectrometer using Al Ka radiation. N_2 _adsorption-desorption measurements were carried out at 77 K using a Quantachrome Autosorb gas-sorption system. Brunauer-Emmett-Teller (BET) and Barrett-Joyner-Halenda (BJH) models were used to determine the specific surface areas and the pore sizes of the samples, respectively. Morphological and structural information were obtained from high-resolution transmission electron microscopy (TEM, JEOL 2010F). Scanning transmission electron microscopy (STEM) was performed on an ARM 200F transmission electron microscope (JOEL; Double spherical aberration correction) operating at 200 kV. STEM images were recorded with a High-angle-annular-dark-field (HAADF) detector (70–250 mrad) and an annular-bright-field (ABF) detector (12–25 mrad).

### Li-O_2_ Cell Assembly and Tests

The CoO electrode (typically 1.0 mg cm^−2^) was prepared by mixing 40 wt % CoO with 40 wt % Super P and 20 wt % nafion (as binders). Carbon electrode was mixed 80 wt % Super P with 20 wt % nafion. As a fair comparison, Co_3_O_4_ electrode was made by mixing 40 wt % Co_3_O_4_ with 40 wt % Super P and 20 wt % nafion. The samples were mixed homogeneously into paste and coated on nickel foam pieces of 0.5 cm × 0.5 cm. For deep discharge-charge performance tests and XRD analysis, around 4.0 mg cm^−2^ of cathodes were prepared. Electrochemical experiments were carried out by using a swagelok cell with a hole drilled only on the cathode to enable O_2_ to flow in. The Li-O_2_ cells were assembled inside the glove box under argon atmosphere (<1 ppm H_2_O and O_2_) by using a clean lithium metal disk (10 mm diameter) as anode, a glass-fiber and a polypropylene (Celgard 2400) as separators, 1 M LiTFSI/TEGDME as electrolyte. Galvanostatic discharge-charge experiments were tested in a LAND battery testing system. For the analysis of gaseous products generated during the charging step, the test cells were first discharged in O_2_ atmosphere, and then the oxygen was completely flushed out by ultra-high-purity Ar. A gas sample was taken from the cell to make a baseline before the charging process. Gas samples for the background analysis and generated during the charging step were analyzed by gas chromatography-mass spectrum (GC-MS, ITQ1100, Thermo Fisher).

## Supplementary Material

Supplementary Information Supplementary Information

## Figures and Tables

**Table 1:** Analysis of gas products after first charge using GC-MS for Li-O_2_ batteries based on Super P and CoO cathodes

	O_2_	CO_2_	O_2_/CO_2_
SP	14.40%	0.64%	22.5
CoO	21.14%	0.35%	59.77
