# Supplementary material for: Compatible interface design of CoO-based Li-O2 battery cathodes with long-cycling stability
Source: Sci Rep. 2015 Feb 27;5:8335. doi: 10.1038/srep08335 (PMC4342555; doi:10.1038/srep08335)
Supplement: Supplementary Information [file srep08335-s1.docx]

**Compatible interface design of CoO-based Li-O_2_ battery cathodes with long-cycling stability**

Chaoqun Shang^ab+^, Shanmu Dong^a+^, Pu Hu^ab^, Jing Guan^a^, Dongdong Xiao^c^, Xiao Chen^a^, Lixue Zhang^a^, Lin Gu^c*^, Guanglei Cui^a*^, Liquan Chen^ac^

a Qingdao Industrial Energy Storage Research Institute, Qingdao Institute of Bioenergy and Bioprocess Technology, Chinese Academy of Sciences, Qingdao 266101, P. R. China

b University of Chinese Academy of Sciences, Beijing 100049, P. R.China

c Institute of Physics, Chinese Academy of Sciences, Beijing 100080, P. R. China

^+^ These authors contributed equally to this work.

* Corresponding Author.

Tel: (+86) 532-80662746. E-mail: [cuigl@qibebt.ac.cn](mailto:cuigl@qibebt.ac.cn)

Tel: (+86) 10-82649550. Email: [l.gu@iphy.ac.cn](mailto:l.gu@iphy.ac.cn)

Figure S1. The cycling performance (a) and coulombic efficiency (b) of Li-O_2_ battery electrode composed of Super P at the current density of 0.04 mA cm^-2^ with deep discharge-charge.


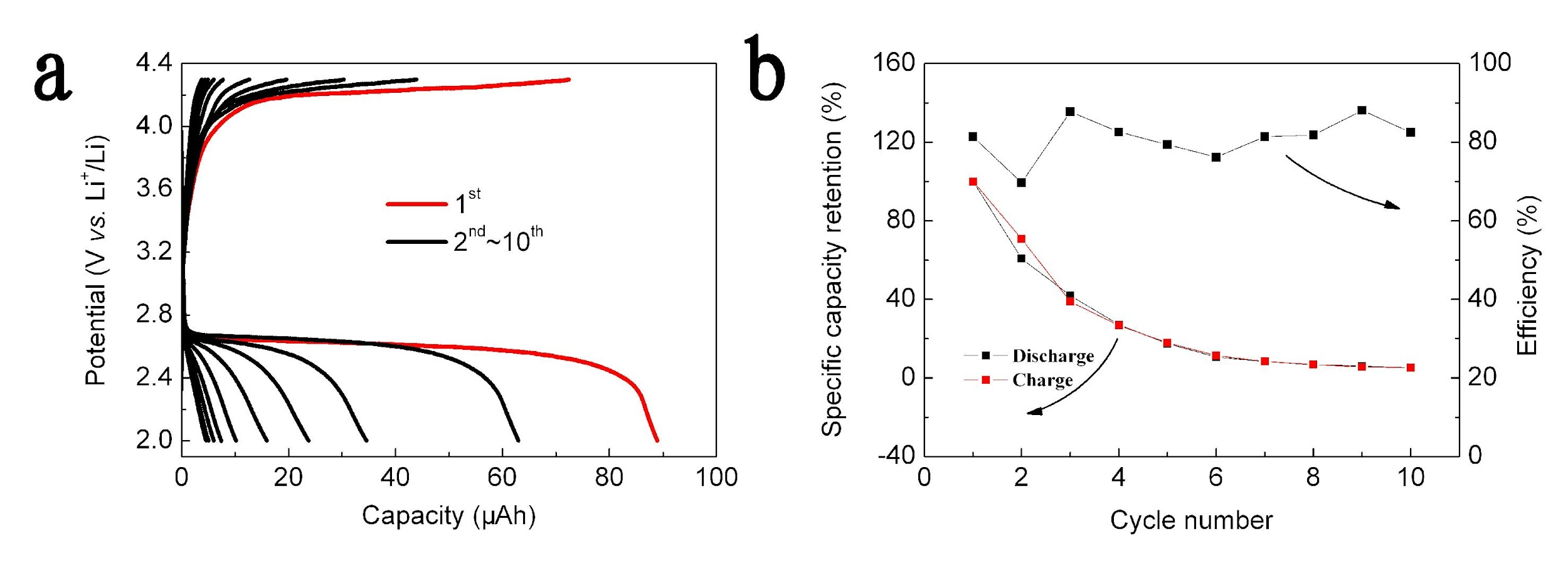


Figure S2. The cycling performance of CoO cathodes at the current density of 0.04 mA cm^-2^ with fixed discharge-charge capacities of 800 mAh g_c_^-1^ (lithium anode: diameter 10 mm; thickness 0.2 mm).


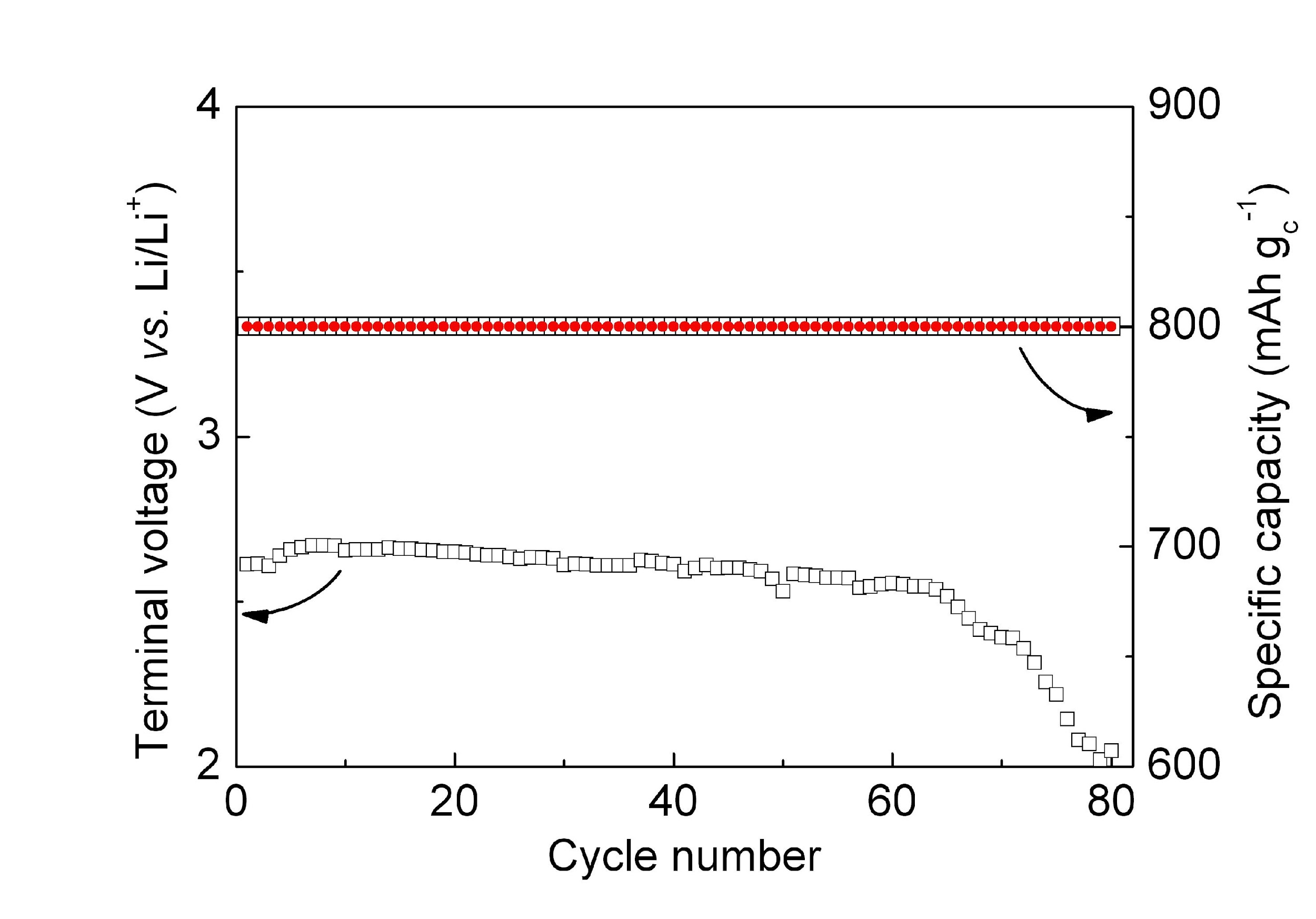


Figure S3. Typical SEM (a) and XRD patterns (b) of Co_3_O_4_. (c) Nitrogen adsorption and desorption isotherms of as-prepared Co_3_O_4_ and their pore-size distribution (inset). (d) The deep discharge-charge cycling performance and coulombic efficiency (inset) of Co_3_O_4_-based electrode Li-O_2_ battery.


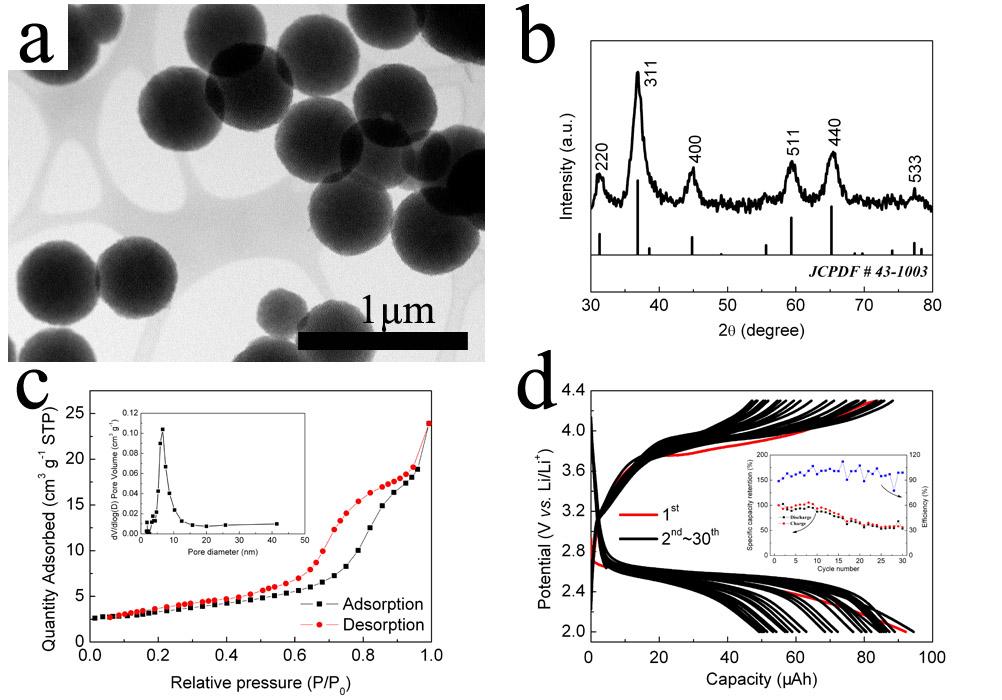


Figure S4. Top views of the optimized geometric structures of pristine graphite (a); side views of the optimized geometric structures of the ideal Co_3_O_4_ (110) surfaces (b); CoO (111) surface models terminated by Co atoms, which is denoted as Co-CoO (111) (c); CoO (111) surface models terminated by O atoms, which is denoted as O-CoO (111) (d); CoO (200) surface (e), and CoO (110) surface (f). The top and bottom layer of carbon atoms are represented by black and light gray balls, Co and oxygen atoms are represented by blue and red balls, respectively.


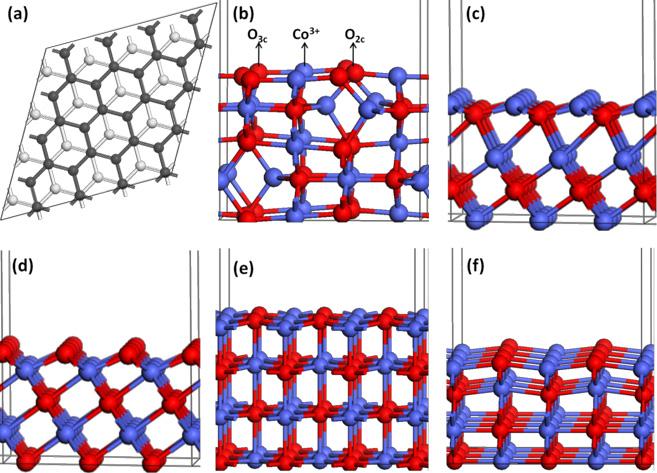


Figure S5. Top views of the most stable adsorption configuration of LiO_2_ on graphite.


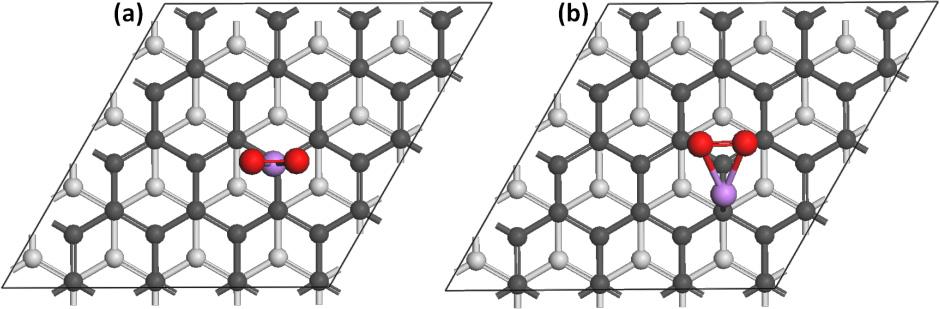


S6 Density functional theory calculations

1. Surface Models

The Super P carbon black is comprised of one-dimensional chain-like structure of interconnected carbon particles. Within each particle, a carbon-like nanostructure composed mainly of *sp*^2^-hybridized carbon atoms is observed. In this study, the graphite model is used to mimic the frameworks of Super P carbon black and systematically investigate the adsorption of LiO_2_ onto the Super P surface. As described in Fig. S4a, the pristine graphite surface was modeled with a 4×4 hexagonal supercell containing two layers of graphene. The graphite sheet was basically composed of 64 carbon atoms. To simulate the edge-functionalized graphite structures, the bilayer-graphite model was decorated with one single hydroxyl group attached to a carbon along one zigzag edge. The constructed supercells were periodically repeated with a 15 Å vacuum region lying vertically, which should be large enough to avoid interplanar interactions.

The Co_3_O_4_ (110) plane was constructed because this surface was reported to be one of the frequently exposed surfaces of Co_3_O_4_, Co_3_O_4_ has two Co^3+^ cations at the octahedral sites and two twofold and two threefold coordinated O^2-^ anions (O2c and O3c) in surface slab model (as shown in Fig. S4b). The slab consists of five atomic layers of Co-O with the bottom three layers kept frozen, while the remaining layers were allowed to relax. A vacuum spacing of about 15 Å was being introduced along consecutive slabs to ensure negligible interaction between cobalt oxide and its periodic images.

We constructed slab models of the CoO surface with the (111), (200) and (110) orientations. As shown in Fig. S4c and S4d, the CoO (111) surface can be either Co- or O-terminated based on the different ways of cleaving a surface from the same bulk-like structure. Each slab is composed of five atomic layers in a (3×3) supercell configuration. The computed models representing CoO (200) surface was modeled by a (3×2) periodic slab of four atomic layers (Fig. S4e). Similarly, a (3×2) four-layer slab was used to model the CoO (110) surface, as illustrated in Fig. S3f. All the slabs were repeated periodically with a 15 Å vacuum spacing between the images in the direction of the normal surface. During geometry optimizations, only the top two atomic layers were allowed to fully relax, while the rest of the atoms were kept fixed at their converged bulk positions.

2. Computational Method

The periodic, self-consistent DFT calculations were performed by using the plane-wave technique as implemented in the Vienna ab initio simulation package (VASP). Projector-augmented waves (PAW) were used to describe the ion cores, and the exchange-correlation interactions were expressed with a generalized gradient approximation (GGA) in the form of Perdew-Wang (PW91) functional. A similar approach was successfully used for the structural calculations of Co_3_O_4_. The plane-wave basis set was expanded to a cutoff energy level of 400 eV. The convergence criteria for the self-consistent electronic iterations and ionic force relaxations were 10^-4^ eV and 0.03 eV/Å, respectively. To save the computational demand, the Brillouin zone integrations were performed with a 3×4×1 *k*-point mesh generated by the Monkhorst-Pack scheme for Co_3_O_4_ and CoO surfaces, however, on graphite surface, a 4×4×1 *k*-point mesh was employed. Ionic relaxation was executed with the conjugated gradient method. Gaussian smearing was used with a smearing parameter of 0.2 eV for these calculations. Throughout the calculations, the spin-polarized approach was performed.

The adsorption energy of LiO_2_ on the surfaces is defined as the energy of LiO_2_ adsorbed on the surface minus the energy sum of the clean surface and the isolated LiO_2_ molecule in a large cubic box (at 15 Å on each side). The adsorption process is exothermic if the adsorption energy is negative.

3. DFT results

The most stable configuration found in the calculations for the pristine graphite is depicted in Fig. S5a. It is found that the top site of the carbon atom is the most preferred position for perpendicular orientations of LiO_2_, with the Li-end readily sitting above the carbon through a single connection. This gives very weak adsorption energy of -0.1 eV. The weak interaction is mainly attributed to the van der Walls forces between LiO_2_ and the pristine sorbent. However, a positive value of 0.1 eV is obtained when oxygen atom approaches to the graphic carbon. Fig. S4b reveals that LiO_2_ molecule is adsorbed in a tilted configuration with oxygen atoms pointed upward from the graphite plane. Therefore, a configuration with minimal overlap between oxygen atoms of LiO_2_ and carbon slab is preferred.

DFT was also used to calculate the optimum adsorption mode and strength of interaction of LiO_2_ onto various cobalt oxide surfaces, including Co_3_O_4_ (110), Co-CoO (111), O-CoO (111), CoO (200) and CoO (110). The adsorption of molecular LiO_2_ follows some general patterns, in which LiO_2_ oxygen atoms interact with surface Co cations and the Li atom interacts with surface basic O^2-^ sites. However, different interaction modes have been observed for each cobalt oxide.

Co_3_O_4_ (110): The DFT-calculated minimum-energy adsorption geometry for LiO_2_ on Co_3_O_4_ (110) is shown in Fig. 5a. The LiO_2_ forms three connections with the Co_3_O_4_ (110) surface, corresponding to the oxygen end of LiO_2_ located on top of Co^3+^ sites and Li^+^ formed bonds with two equivalent O^2-^ sites, so that the (O-Li-O-O-Co) adopted a distorted pentagonal structure. The resultant adsorption energy of this state is -2.5 eV. The strong adsorption gives rise to the localized geometric changes for the adsorbed LiO_2_ molecule, where one Li-O bond is elongated to 2.49 Å with respect to the corresponding 1.78 Å for the free triangular LiO_2_ molecule.

O-CoO (111): The configuration with highest adsorption energy (-2.7 eV) schematically shown in Fig. 5b, has the LiO_2_ molecule lying almost perpendicular to the CoO (111) surface terminated with O atoms. The Li-end of the molecule is readily linked with three neighboring lattice O^2-^ ions. One of the Li-O bond lengths shows an elongation of 0.81 Å compared to the gas-phase value. In this case, no significant structural variation is observed for O-CoO (111) surface upon adsorption.

CoO (200): The most stable structure has adsorption energy of -4.2 eV and is displayed in Fig. 5c. In the resultant geometry, the LiO_2_ molecule is attached to two neighboring Co^2+^ sites through both O atoms. The Li atom is adsorbed in the vicinity of an O^2-^ ion. The molecular axis evolves to a parallel position to the surface plane. It is seen that the bond lengths of LiO_2_ are affected only little by the adsorption. However, the CoO (200) surface becomes disturbed because of the strong attractive interaction between LiO_2_ oxygen atoms and Co^2+^ cations, which makes Co^2+^ move up from the surface plane.

CoO (110): For the most stable configurations (Fig. 5d), the LiO_2_ molecular axis is slightly tilted toward the CoO (110) surface. Each O atom of the LiO_2_ molecule is calculated to adhere strongly to the supported Co^2+^ sites. Moreover, the Li-end of the molecule sits across two adjacent O^2-^ sites. Accordingly, the chemisorption bond between LiO_2_ and the surface is greatly strengthened, as manifested by the increased adsorption energy of -6.4 eV. This leads to the scission of the O-O bond as indicated by the calculated distance of 3.02 Å.

Co-CoO (111): The DFT-optimized most probable adsorption sites that exist at CoO (111) surface terminated with Co atoms are shown in Fig. 5b. LiO_2_ is vertically attached on three adjacent Co^2+^ sites via each oxygen atom, to give the largest adsorption energy of -13.4 eV. Consequently, the LiO_2_ structure is drastically distorted upon its interaction with these coordinatively unsaturated Co sites. The O-O bond length has been stretched to 2.51 Å, compared to the value of 1.37 Å for triangular LiO_2_ molecule. More interestingly, the interaction of LiO_2_ induces a significant modification on the CoO (111) substrate. The surface Co^2+^ atoms bonded to the O-end of LiO_2_ in this configuration tend to be pulled out of the surface plane in order to ensure the multiple connections with LiO_2_ species.

On the basis of our DFT calculations, it is observed that the exothermicity for LiO_2_ adsorption on various surfaces increases in the order of Co-CoO (111) > CoO (110) > CoO (200) > O-CoO (111) > Co_3_O_4_ (110). It thus appears that the interaction of LiO_2_ with the CoO surfaces is significantly larger than that determined for Co_3_O_4_ (110). The changes in binding strengths with the variation of surfaces can be understood in terms of the distributions of the coordinatively unsaturated Co^2+^ or Co^3+^ adsorption sites. It can be seen that, the CoO (200) and CoO (110) surfaces enable the bonding of LiO_2_ to vicinal Co^2+^ site via two Co-O connections, which serves to stabilize the adsorbed LiO_2_. In the case of Co-CoO (111), there is an increase in the total number of direct Co-O bonds to six. In contrast, the ability to attract LiO_2_ becomes attenuated on Co_3_O_4_ (110), where only one Co^3+^ site is involved in bonding interactions with O atom of LiO_2_. In accord with the experimental observations, we can assume that the main reason for the improved performance of CoO as a cathode material lies in the increased adsorption ability with the adsorbate.

It is noted that the binding of molecular LiO_2_ onto CoO surface is significantly larger than the corresponding value of graphite, from which we conclude that CoO can serve as a good sorbent for LiO_2_ capturer and is considered the promising cathode candidate for Li-O_2_ batteries.
